# Design and analysis of centrifugal compressor in carbon dioxide heat pump system

**DOI:** 10.1038/s41598-024-55698-y

**Published:** 2024-03-04

**Authors:** Peng Jiang, Yong Tian, Bo Wang, Chaohong Guo

**Affiliations:** Institute of Engineering Thermophysics, Chinese Academy of Sciences, 11 North Fourth Ring West Road, Beijing, 100190 People’s Republic of China

**Keywords:** Carbon dioxide heat pump, Carbon dioxide centrifugal compressor, Compressor performance, Axial force, Rotor dynamics, Aerospace engineering, Mechanical engineering

## Abstract

Based on the advantages of energy saving, environmental protection and high efficiency, carbon dioxide heat pump system has great application prospects. However, there are still many technical problems to be solved, especially the design and optimization of carbon dioxide centrifugal compressor. In this paper, a centrifugal compressor in carbon dioxide heat pump system is designed. The compressor is directly driven by a high-speed permanent magnet synchronous motor. Two-stage impellers are installed on both sides of the motor, and the bearings are active magnetic bearings. The influences of inlet pressure and temperature on compressor performance are analyzed. In the range of inlet temperature from 35 to 55 °C, with the decrease of inlet temperature, the compressor pressure ratio increases by 12–29.8%, the power increases by 2.7–8.6%. In the range of inlet pressure from 4 to 6 MPa, with the increase of inlet pressure, the compressor pressure ratio increases by 12.3–38.6%, and the power increases by 8.7–17.8%. In addition, the calculation method of compressor axial force is introduced, the axial force is calculated, analyzed and optimized. Furthermore, the rotor dynamics of compressor rotor and the influences of bearing stiffness and diameter of motor rotor on rotor dynamics are studied. With the increase of bearing stiffness, the first-order critical speed and maximum displacement of the rotor increase. The research provides a theoretical reference for the design and optimization of centrifugal compressor in carbon dioxide heat pump system.

## Introduction

With the development of social economy, human demand for energy continues to increase, and fossil energy still accounts for more than 80% of global energy consumption, which has caused a series of environmental problems. Therefore, it is urgent to find alternative clean and renewable energy^1,2^. In recent years, the utilization modes of new energy sources such as nuclear energy^3,4^, solar energy^5,6^, wind energy^7,8^, biomass energy^9,10^, hydrogen energy^11,12^, geothermal energy^13^ and tidal energy^14^ have been deeply studied.

Similarly, improving energy efficiency is also an important means to save energy and reduce emissions. Through the heat pump effect, the energy of low-temperature heat source can be effectively utilized and the system efficiency can be improved. Natural refrigerants widely used in heat pump systems include carbon dioxide, ammonia, butane, isobutane and propylene^15^. The ozone depletion potential of carbon dioxide is zero, and it is neither explosive nor toxic, and it is cheap, which makes the carbon dioxide heat pump system widely studied^16–22^. Xu et al.^18^ put forward an improved integrated CO_2_ heat pump system, which can improve thermal comfort and reduce economic cost, and can operate in hot water combined mode and space heating mode. Nawaz et al.^19^ developed a performance model to evaluate the characteristics of a transcritical CO_2_ HPWH system, and found that the set value of water supply temperature has a great influence on the system performance. A new pumped thermal energy storage (PTES) structure with supercritical CO_2_ as working fluid and molten salt as heat storage fluid was proposed by Tafur-Escant et al.^20^, and the net power generated by this novel proposal is 12.46 MW in load and 10 MW in discharge, achieving an efficiency of 80.26%. Qin et al.^21^ put forward a new compression/injection transcritical CO_2_ heat pump system for simultaneous cooling and heating, and found that the new system has 12% higher efficiency than the injection system, and produces the shortest payback period of 1.10 years among the three systems considered. Wang et al.^22^ conducted experimental research on the hot gas bypass defrosting method and found that, except for the initial stage, the measured parameters gradually change during the defrosting process, and the defrosting time is within the specified defrosting range.

As the core component of heat pump system, compressor plays an important role in system efficiency. At present, the main types of compressors used for carbon dioxide are piston compressor^23,24^, scroll compressor^25,26^, screw compressor^27^ and centrifugal compressor^28^. In recent years, a lot of research work has been done to improve the performance of carbon dioxide compressor. A model of the thermal compressor was developed and validated by Ibsaine et al.^23^. The simulation results related to the regenerator, piston rod diameter, adiabatic dead volume size, and working fluid leakage in the annular gap between the cylinder liner and piston are provided. Zheng et al.^25,26^ proposed a passive flow control method for the radial leakage problem of vortex compressors using micro grooves at the end of the vortex envelope and continuous sealing grooves on the static vortex sidewall. They conducted numerical research on their flow characteristics and found that the aerodynamic and thermodynamic performance of oil-free vortex compressors can be effectively improved by using micro groove vortex blade tips under reasonable working conditions and micro groove geometry. How the rotor force generated by compression and expansion processes is partially balanced to reduce radial bearing force and eliminate axial force was demonstrated by Stosic et al.^27^.

Centrifugal compressor has the advantages of small volume, light weight and compact structure. The design method, performance and application effect in carbon dioxide cycle have also been studied by scientists^28–33^. A a mean line model for centrifugal compressors and a coupling optimization method with heat pump systems, and coupled and optimized the compressor model with a heat pump cycle model supplying steam at 150 °C are proposed by Meroni et al.^28^. Cao et al.^29^ proposed a design strategy to improve the aerodynamic performance of supercritical CO_2_ centrifugal compressors and suppress condensation, and found that increasing the inlet compression coefficient or density within the studied operating conditions would reduce the condensation area. A 150 kW supercritical CO_2_ centrifugal compressor for a 500 kW power generation system was designed and studied by Park et al.^30^. The design point, pressure ratio, and efficiency of the compressor are 1.75 and 80%, respectively, with a speed of 36000 RPM. Hosseinpour et al.^31^ designed a single stage s-CO_2_ centrifugal impeller for simple recovery of s-CO_2_ in the Brayton cycle. A new set of design guidelines for s-CO_2_ compressors from zero-dimensional, one-dimensional, and three-dimensional perspectives were proposed by Xu et al.^32^. Xia et al.^33^ proposed and simulated a simple regeneration cycle of 5 MW s-CO_2_ for a small lead cooled fast reactor (SLFR) for the first time, and designed an s-CO_2_ compressor for this cycle.

At present, there is little research on the design and application of centrifugal compressor in carbon dioxide heat pump, especially the centrifugal compressor with magnetic bearings and permanent magnet synchronous motor, which has the advantages of compact structure, oil-free, non-leakage and high efficiency. Because of the high working pressure of carbon dioxide heat pump, centrifugal compressor, as the core component of heat pump, puts forward higher requirements for its axial force optimization. In this study, the axial force of the whole machine is optimized to control it in a reasonable range. At the same time, the compressor structure (CMC structure) which directly drives one impeller on both sides of the high-speed motor is adopted in this study, which increases the length of the motor rotor, which poses a greater challenge to the check of rotor dynamics. Therefore, the design and optimization of compressor in this study are of great significance to the popularization and use of carbon dioxide heat pump.

**Figure 1:**
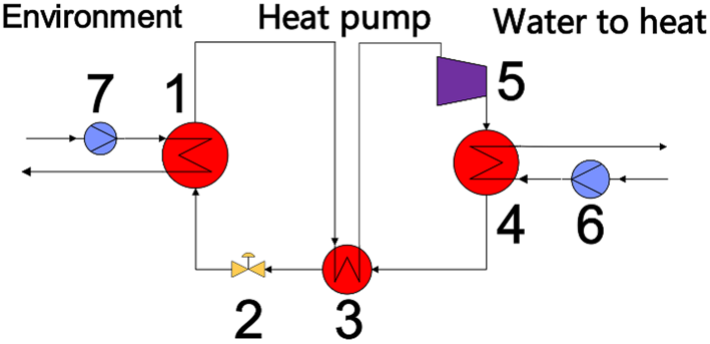
Schematic diagram of carbon dioxide heat pump system. 1, 3, 4. Heat exchanger; 2. Control valve; 5. Compressor; 6, 7. Pump.

The carbon dioxide heat pump system is built in Lianyungang City, Jiangsu Province, as an auxiliary system of High-Efficiency Low-Carbon Gas Turbine Test Device, which is mainly used for waste heat recovery in hot water. The schematic diagram of carbon dioxide heat pump system is shown in Fig. 1. The temperature of the low temperature heat source is 40 °C, the heating capacity of the system is greater than 1 MW, and the external water supply temperature is higher than 70 °C. This article introduces the preliminary design of single-shaft back-to-back CO_2_ centrifugal compressor used in CO_2_ heat pump system, introduces the calculation and optimization method of axial force of CO_2_ centrifugal compressor, and analyzes the influences of magnetic bearing stiffness and diameter of motor rotor on rotor dynamics.

## Rotor layout

**Figure 2:**
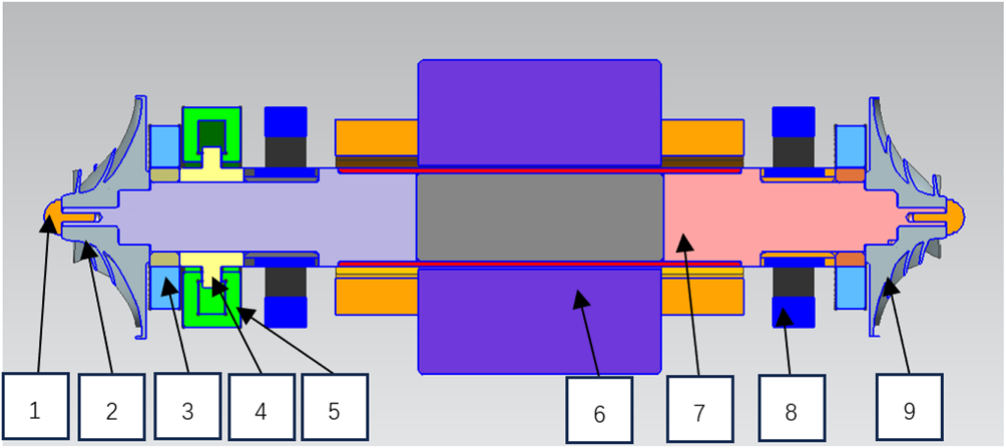
Layout of carbon dioxide compressor.

The layout of the carbon dioxide compressor is shown in Fig. 2, and the descriptions of each component are as follows.

1. Nut
2. First Stage Impeller
3. Seal Structure
4. Thrust Collar
5. Thrust Bearing
6. Motor Stator
7. Motor Rotor
8. Radial Bearing
9. Second Stage Impeller

The carbon dioxide centrifugal compressor will be designed according to API 617^34^. Nuts are used to fix the rotor in the axial direction, and the impeller and the motor cavity are sealed by a sealing structure. The motor adopts a permanent magnet synchronous motor, and the compressor impeller is installed on both sides of the motor shaft and directly driven by the motor. Comb seal or dry gas seal can be selected as the sealing structure according to the design requirements. Both the radial bearing and the thrust bearing adopt active magnetic suspension bearings, which have long service life and do not need a lubricating oil system. Most axial force can be offset by the back-to-back design of the first-stage impeller and the second-stage impeller, and the balance of residual axial force in the whole rotor system is finally realized by the thrust bearing.

## Compressor sizing

According to the conditions of carbon dioxide heat pump cycle, the operating conditions of the compressor are determined as shown in Table 1. The aerodynamic design of compressor is completed using Compal software^35^. The first-stage design pressure ratio is 1.6, and the second-stage design pressure ratio is 1.43. The parameters of the two-stage impellers are shown in Table 2.

**Table 1:** Operating conditions for compressor design.

Parameter	Compresssor
Inlet pressure P_1_/MPa	4.95
Inlet temperature T_1_/°C	45
Inlet mass flow rate/kg s^−1^	≥ 6
Outlet pressure P_2_/MPa	11
Max speed/rpm	26000

**Table 2:** Parameters of impellers.

Parameter	First stage	Second stage
Impeller-hub inlet diameter/mm	26.0	23.2
Impeller exit diameter/mm	133	130.3
Impeller exit width/mm	3.93	3.64
Clearance gap/mm	0.3	0.3
Number of impeller main blades	7	7
Number of impeller splitter blades	7	7
Vaneless diffuser exit diameter/mm	172.9	169.4

**Figure 3:**
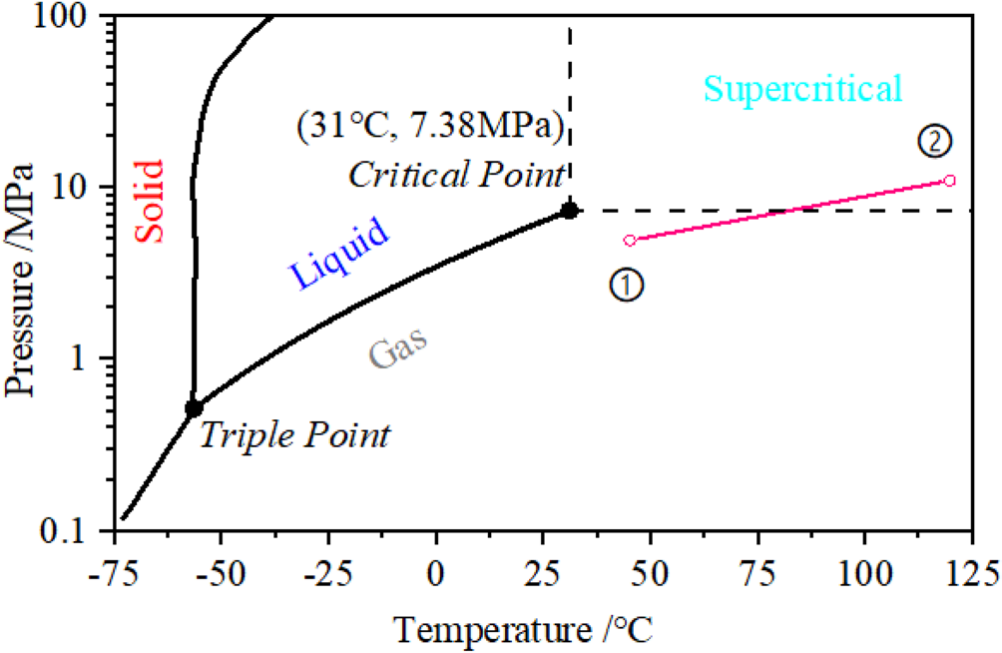
Carbon dioxide three-phase distribution diagram.

The three-phase distribution diagram of carbon dioxide is shown in Fig. 3, and the Inlet state of compressor (point 1) and outlet state (point 2) are identified in Fig. 3. Carbon dioxide at the inlet of the compressor is in a gaseous state, and the carbon dioxide at the outlet after being compressed by the impellers is in supercritical state, so the whole compression process is transcritical.

**Figure 4:**
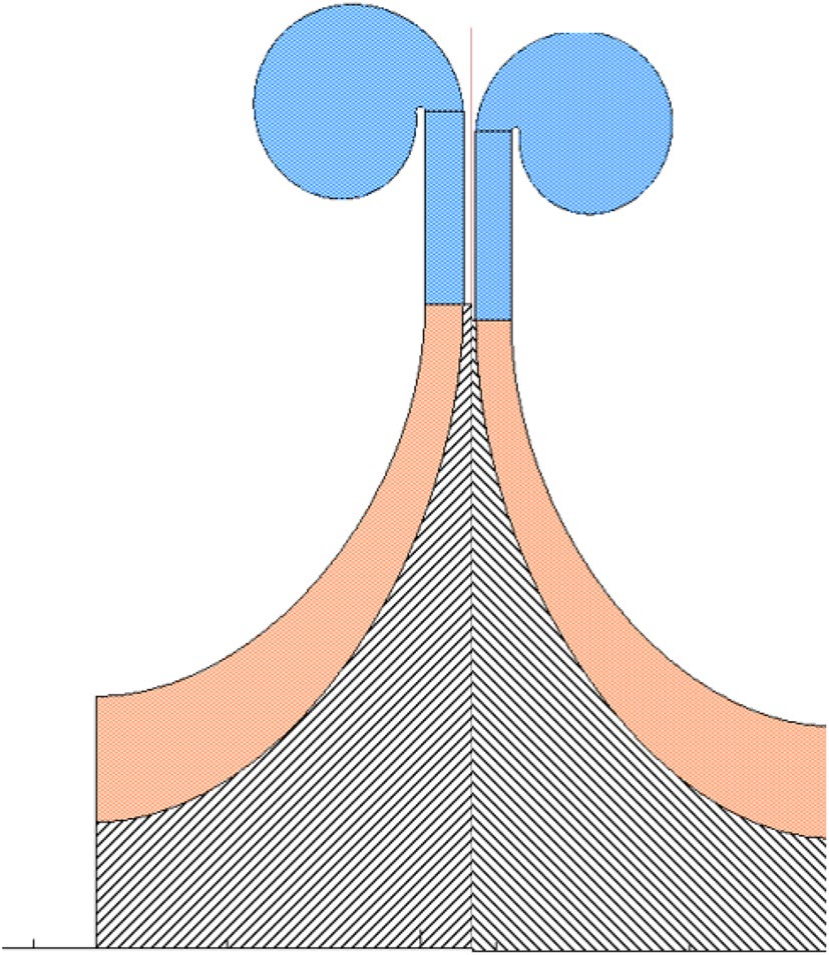
Layout of the Two-Stage Compressor Design.

The layout of the two-stage centrifugation compressor design is shown in Fig. 4. A diffuser and a volute are designed behind each stage impeller, and the outlet of the first stage volute is connected with the inlet of the second stage impeller through an external pipeline.

**Figure 5:**
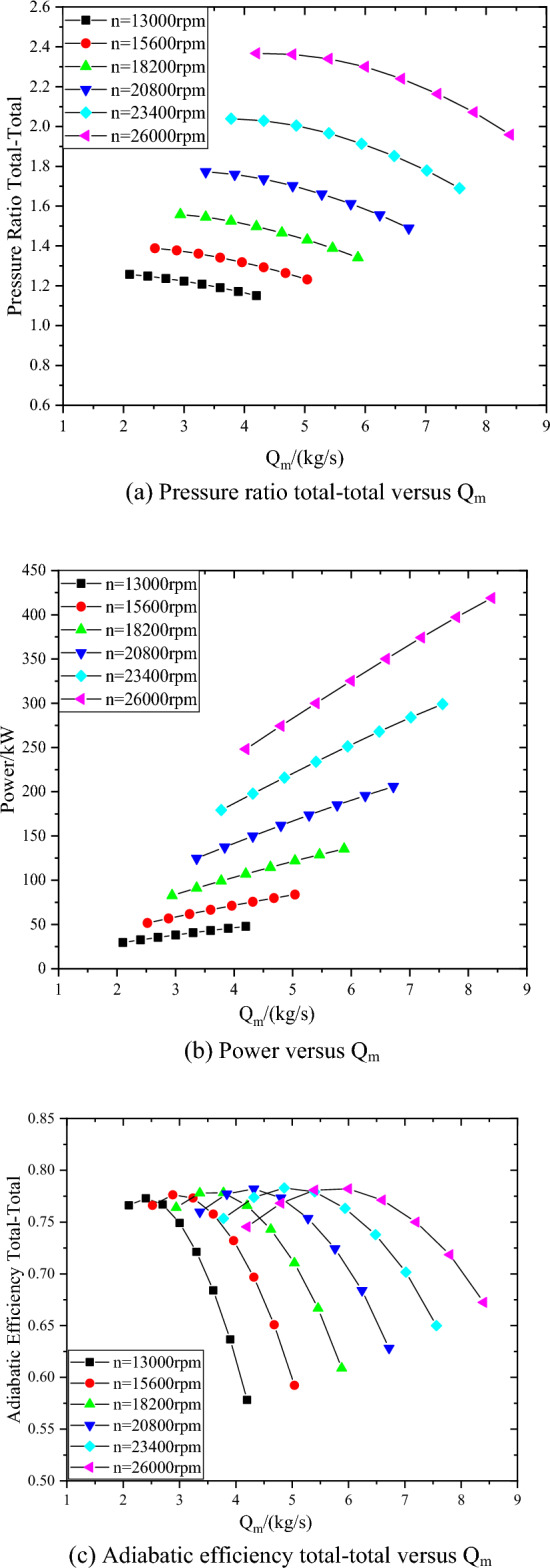
Performance curves of the compressor at 13000–26000 rpm.

The performance of compressor can be accurately predicted by Compal software. The performance curves of the compressor are shown in Fig. 5. With the increase of flow rate, the pressure ratio of compressor decreases, the power increases, and the efficiency first increases and then decreases. With the increase of rotating speed, the pressure ratio and power of the compressor gradually increase. At low speed, the efficiency of compressor changes more sharply with the flow rate. The main reason is that the working point deviates greatly from the rated design point when the compressor is running at low speed.

**Figure 6:**
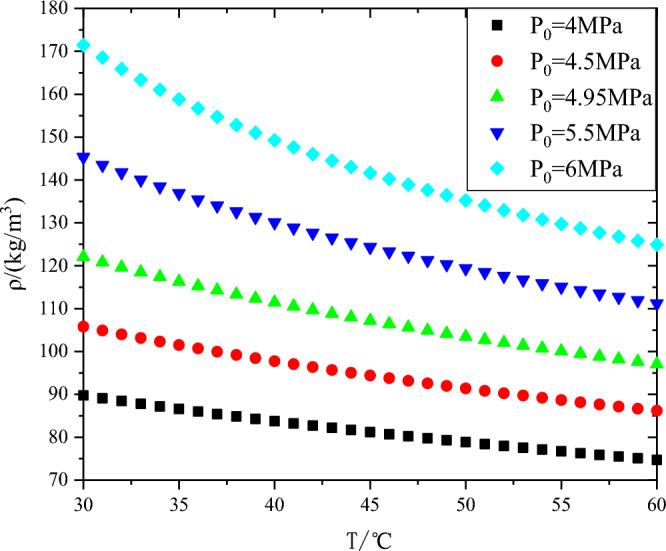
Variation diagram of carbon dioxide density with temperature and pressure.

Because carbon dioxide is gaseous at the compressor inlet, as shown in Fig. 6, the density of carbon dioxide is greatly influenced by temperature and pressure. When the temperature is 45 °C and the pressure increases from 4 to 6 MPa, the density of carbon dioxide increases by 74.4%. The density of carbon dioxide increased by 20.5% when the pressure was 4.95 MPa and the temperature was increased from 30 to 60 °C. In the carbon dioxide heat pump cycle, the inlet parameters will also change with the external parameters when the compressor is running, so it is of great significance to study the influence of compressor inlet parameters on compressor performance for compressor control in the cycle.

**Figure 7:**
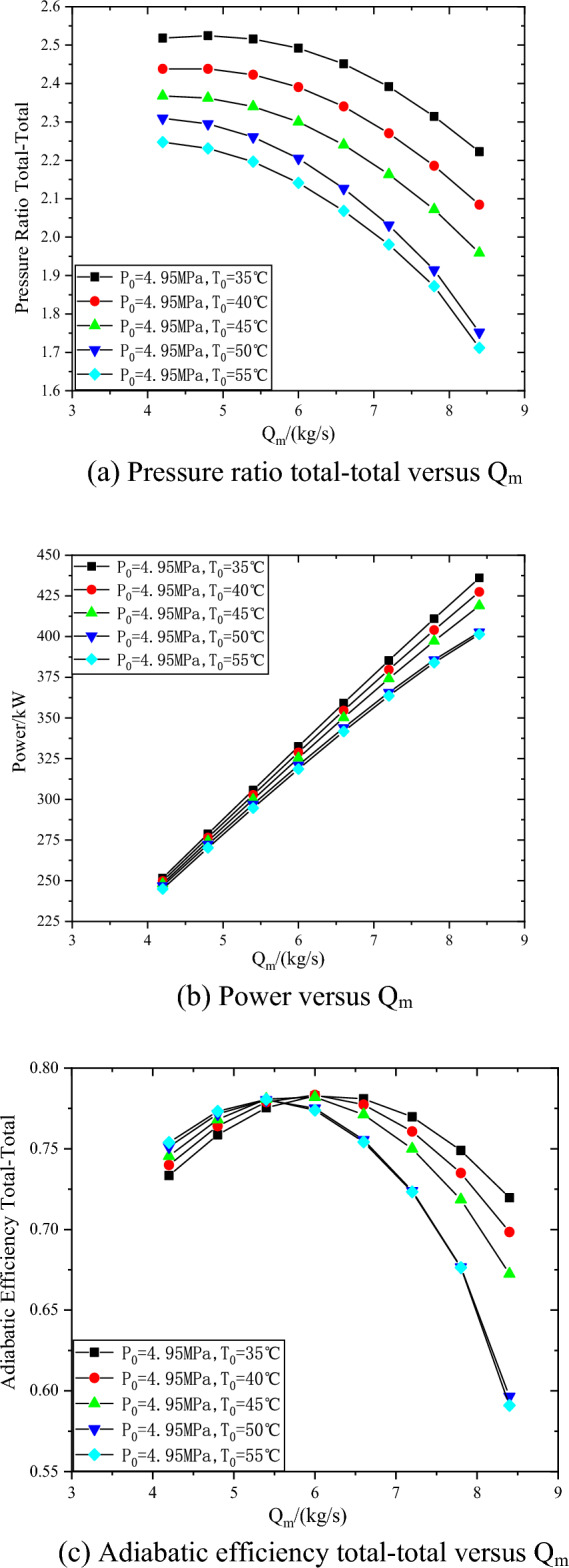
Performance curves of the compressor at 26000 rpm with different inlet temperatures.

The predicted performance curves of the compressor at 26000 rpm, inlet pressure of 4.95 MPa and inlet temperature from 35 to 55 °C are shown in Fig. 7. In the range of inlet temperature from 35 to 55 °C, with the decrease of inlet temperature, the compressor pressure ratio increases by 12–29.8%, the power increases by 2.7–8.6%, and the efficiency decreases more slowly after the design point. This is mainly because, at the same inlet pressure, with the decrease of temperature, the density of carbon dioxide increases, and the volume flow at the inlet of the compressor decreases, so that a larger pressure ratio can be achieved at the same speed, and similarly, the compressor power will also increase.

**Figure 8:**
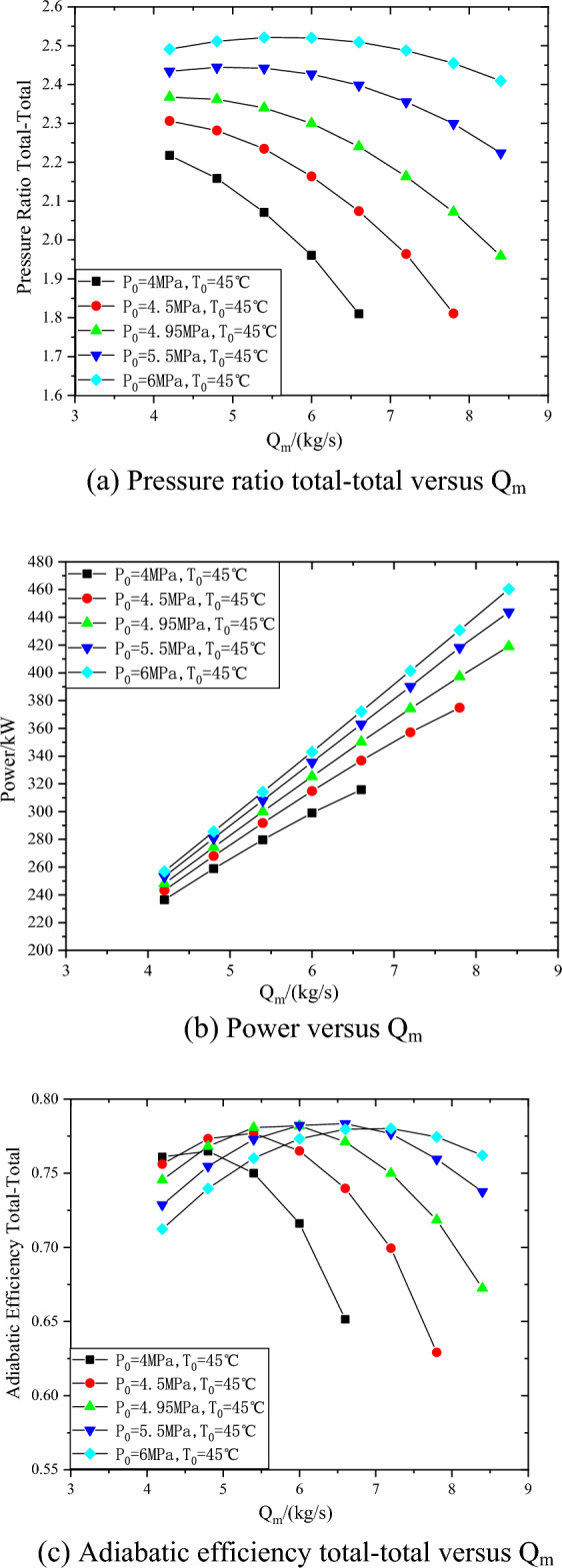
Performance curves of the compressor at 26000 rpm with different inlet pressures.

The predicted performance curves of the compressor at 26000 rpm, inlet temperature of 45 °C and inlet pressure from 4 to 6 MPa are shown in Fig. 8. In the range of inlet pressure from 4 to 6 MPa, with the increase of inlet pressure, the compressor pressure ratio increases by 12.3–38.6%, and the power increases by 8.7–17.8%. The maximum inlet mass flow of compressor at inlet pressure of 4 MPa is 21.4% lower than that at inlet pressure of 6 MPa. With the increase of pressure, the density of carbon dioxide increases, the volume flow at the inlet of compressor decreases and the pressure ratio increases. When the inlet pressure of the compressor is 6 MPa, the pressure ratio of the compressor changes relatively little with the flow rate, mainly because the higher the inlet pressure, the greater the carbon dioxide density, and the smaller the change of the inlet volume flow rate in the same mass flow range.

## Axial force analysis

The centrifugal compressor in carbon dioxide heat pump cycle has high pressure and high density, which leads to a very large axial force of a single impeller and requires high design requirements for axial thrust bearings. Therefore, it is necessary to accurately predict and optimize the axial force of the compressor to ensure its safe and stable operation. The axial force of carbon dioxide compressor is related to many parameters, such as flow rate, sealing diameter, impeller size, impeller inlet and outlet pressure, temperature and so on. In the design process of carbon dioxide compressor, the calculation method of axial force of compressor is as follows.

**Figure 9:**
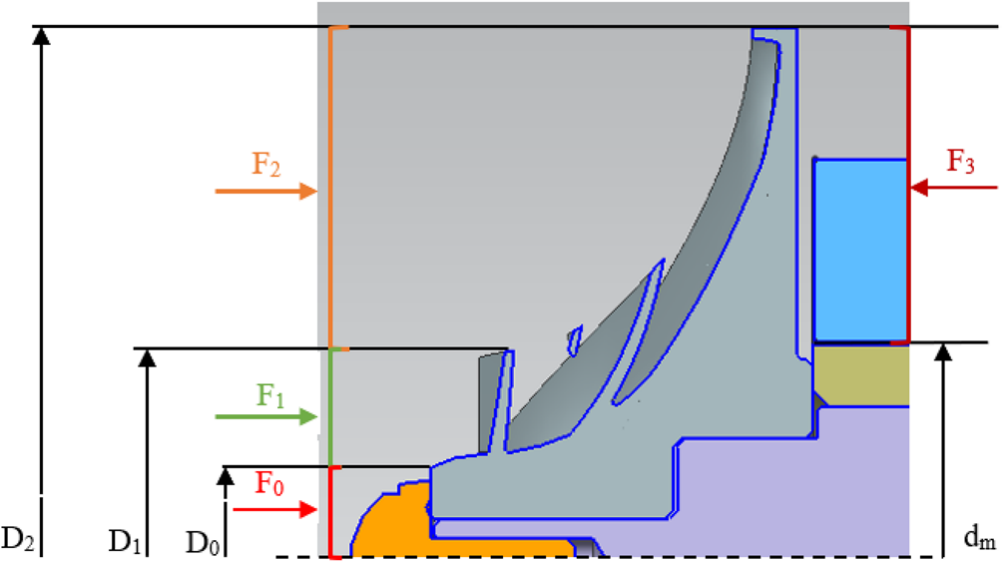
Axial force diagram of impeller.

As shown in Fig. 2, the two-stage impellers of the compressor designed in this study are set back to back, and the axial force directions of the two impellers are opposite, and the axial force of the compressor is as shown in Eq. (1). As shown in Fig. 9, the axial force of the single-stage impeller of the compressor is mainly divided into four parts. The gas force F_0_ acts on the inlet hub of the compressor impeller, the gas force F_1_ acts on the inlet surface, the gas force F_2_ acts on the inlet–outlet part of the impeller, and the gas force F_3_ acts on the back part of the impeller from the sealing diameter to the outer diameter of the impeller. The relationship between the parts is as follows.

1 $${F}_{tot}={F}_{tot1}-{F}_{tot2}$$

2 $${F}_{tot\mathrm{1,2}}={F}_{3}-{F}_{2}-{F}_{1}-{F}_{0}$$

3 $${F}_{0}=\frac{\pi }{4}{D}_{0}^{2}{p}_{0}$$

4 $${F}_{1}={F}_{10}+{F}_{1m}$$

where F_tot_ is the axial force of compressor, F_tot1_ is the axial force of first-stage impeller, F_tot2_ is the axial force of second-stage impeller, F_tot1,2_ is the axial force of the first-stage impeller or the second-stage impeller, D_0_ is the diameter of the impeller inlet hub, and p_0_ is the pressure in front of the impeller inlet hub. Where F_10_ is the axial force generated by the static pressure of inlet gas, and F_1m_ is the axial force generated by the change of momentum from axial direction to radial direction^36^. The calculation formulas are as follows:

5 $${F}_{10}=\frac{\pi {(D}_{1}^{2}-{D}_{0}^{2})}{4}{p}_{1}$$

6 $${F}_{1m}={Q}_{m}{c}_{01}$$

7 $${F}_{1}=\frac{\pi {(D}_{1}^{2}-{D}_{0}^{2})}{4}{p}_{1}+{Q}_{m}{c}_{01}$$

8 $${F}_{2}={\int }_{{r}_{1}}^{{r}_{2}}{p}_{r2}\cdot 2\pi rdr$$

where D_0_ is the diameter of impeller inlet hub, D_1_ is the diameter of impeller inlet, p_1_ is the inlet pressure of impeller, Q_m_ is the inlet mass flow rate, ${c}_{01}$ is the inlet velocity,

Suppose that the change rule of p is as follows.

9 $${p}_{r2}={{p}_{1}+(p}_{2}{-{p}_{1})\left(\frac{r-{r}_{1}}{{r}_{2}-{r}_{1}}\right)}^{2}$$

where p_2_ is the outlet pressure of impeller, r_1_ is the radius of impeller inlet, r_2_ is the radius of impeller outlet.

The following relation is obtained:

10 $${F}_{2}={\int }_{{r}_{1}}^{{r}_{2}}{p}_{r2}\cdot 2\pi rdr=\frac{\pi }{4}\left({D}_{2}^{2}-{D}_{1}^{2}\right){p}_{1}-\frac{8\pi ({p}_{2}{-p}_{1})}{{({D}_{2}{-D}_{1})}^{2}}\left[\frac{{D}_{2}^{4}}{64}-\frac{{D}_{1}^{4}}{196}-\frac{{D}_{2}^{3}{D}_{1}}{24}+\frac{{D}_{2}^{2}{D}_{1}^{2}}{32}\right]$$

where D_2_ is the diameter of impeller outlet.

The axial thrust formula on the back of the impeller from the sealing diameter to the outer diameter of the impeller can be expressed as follows:

11 $${F}_{3}=2\pi {\int }_{{r}_{m}}^{{r}_{2}}{p}_{r3}rdr=\frac{\pi }{4}\left({D}_{2}^{2}-{d}_{m}^{2}\right){p}_{2}-\frac{\pi \rho {u}_{2}^{2}}{32}\left({(D}_{2}^{2}-{d}_{m}^{2}\right)-{\frac{1}{{2D}_{2}^{2}}(D}_{2}^{4}-{d}_{m}^{2}))$$

where d_m_ is the diameter of the sealing diameter between the back of the impeller and the motor.

The related dimension parameters of axial force are shown in Table 3.

**Table 3:** Dimension parameters.

	First stage	Second stage
D_0_/mm	26	23.2
D_1_/mm	52	46.4
D_2_/mm	133	130.3

**Figure 10:**
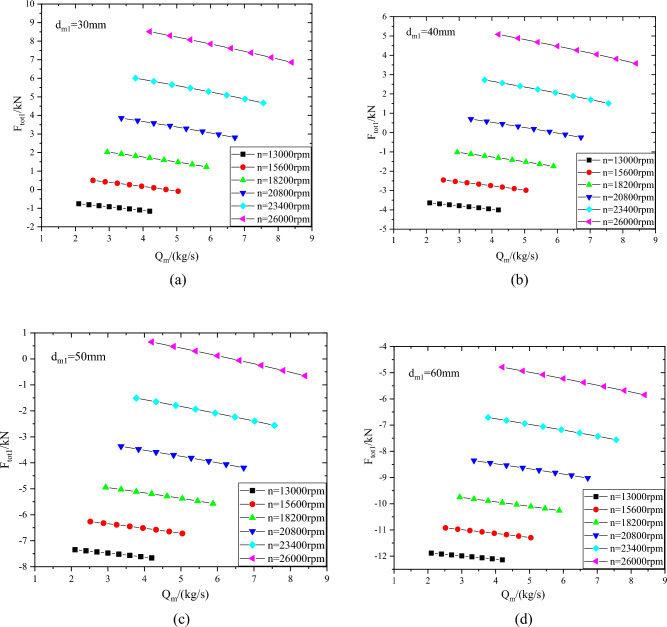
Axial force of first-stage impeller with different sealing diameters (**a**) d_m1_ = 30 mm, (**b**) d_m1_ = 40 mm, (**c**) d_m1_ = 50 mm, (**d**) d_m1_ = 60 mm.

As shown in Fig. 10, the axial force of the first-stage impeller with different sealing diameters d_m1_. With the increase of rotating speed, the axial force direction of the first-stage impeller changes from the reverse direction (inlet flow direction) to the positive direction. Due to the increase of rotating speed, the outlet pressure of the impeller increases and the gas force F_3_ in Eq. (2) increases. With the increase of mass flow rate, the reverse value of axial force of the first-stage impeller increases and the positive value decreases, mainly because the gas force F_1_ in Eq. (2) increases and the axial force value of the impeller decreases with the increase of mass flow rate. In the range of dm1 from 30 to 60 mm, with the increase of the diameter of the sealing diameter, the positive value of the axial force of the impeller decreases, the reverse value increases and the variation range of the axial force decreases. In the range of rotating speed from 13000 to 26000 rpm, the axial force varies from  − 1.16kN to 8.51kN when sealing diameter d_m1_ is 30 mm, and from  − 12.14kN to  − 4.79kN when sealing diameter d_m1_ is 60 mm.

**Figure 11:**
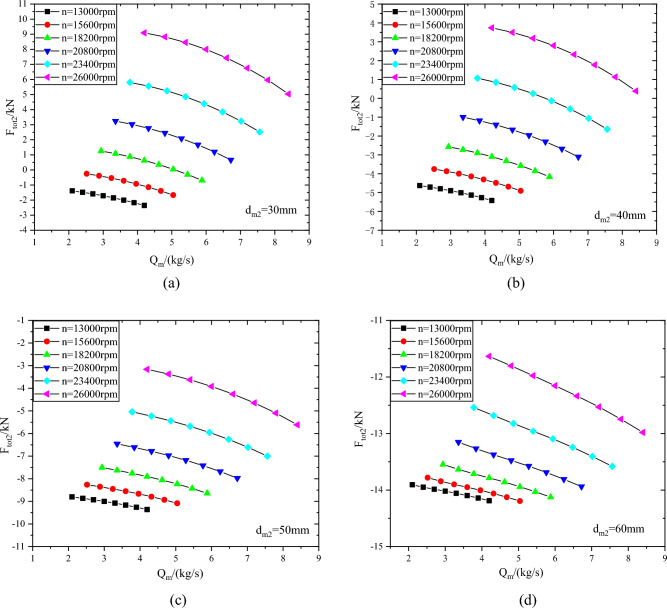
Axial force of second-stage impeller with different sealing diameters (**a**) d_m2_ = 30 mm, (**b**) d_m2_ = 40 mm, (**c**) d_m2_ = 50 mm, (**d**) d_m2_ = 60 mm.

As shown in Fig. 11, the axial force of the second-stage impeller with different sealing diameters d_m2_. The axial force variation law of the second-stage impeller is basically the same as that of the first-stage impeller. In the range of rotating speed from 13000 to 26000 rpm, when the sealing diameter d_m2_ is 30 mm, the axial force of the second-stage impeller changes from  − 2.36kN to 9.09kN, and when the sealing diameter d_m2_ is 60 mm, the axial force changes from  − 14.19kN to  − 11.63kN. When the sealing diameter d_m2_ is 60 mm, the axial force of the second-stage impeller has a smaller range than that of the first-stage impeller.

**Figure 12:**
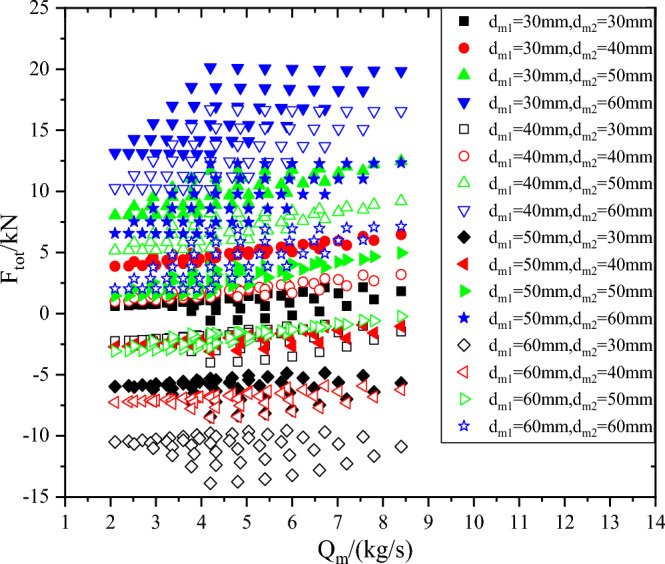
Axial force of compressor.

In the range of sealing diameter d_m_ from 30 to 60 mm, the axial force of compressor is shown in Fig. 12. When the first-stage impeller sealing diameter d_m1_ is constant, the positive value of the axial force of the compressor increases and the negative value decreases when the second-stage impeller sealing diameter d_m2_ ranges from 30 to 60 mm. When the sealing diameter of the second-stage impeller d_m2_ is constant, the positive value of the axial force of the compressor decreases and the negative value increases when the sealing diameter of the first-stage impeller d_m1_ ranges from 30 to 60 mm. Therefore, it is necessary to find two suitable sealing diameters d_m1_ and d_m2_ to keep the axial force of the compressor in a suitable fluctuation range and reduce the design difficulty of the thrust bearing.

**Figure 13:**
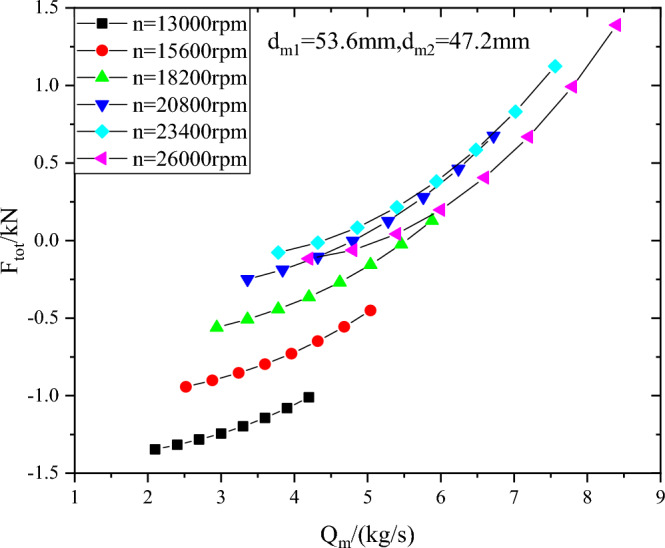
Axial force of compressor when d_m1_ = 53.6 mm and d_m2_ = 47.2 mm.

Combined with the motor rotor size, and through the optimization of the first-stage seal diameter d_m1_ and the second-stage seal diameter d_m2_, it is found that when d_m1_ = 53.6 mm and d_m2_ = 47.2 mm, the axial force of the compressor is within ± 1.5kN, and the absolute value and variation range of the axial force are the best. The relationship between the axial force and the flow rate and speed is shown in Fig. 13.

**Figure 14:**
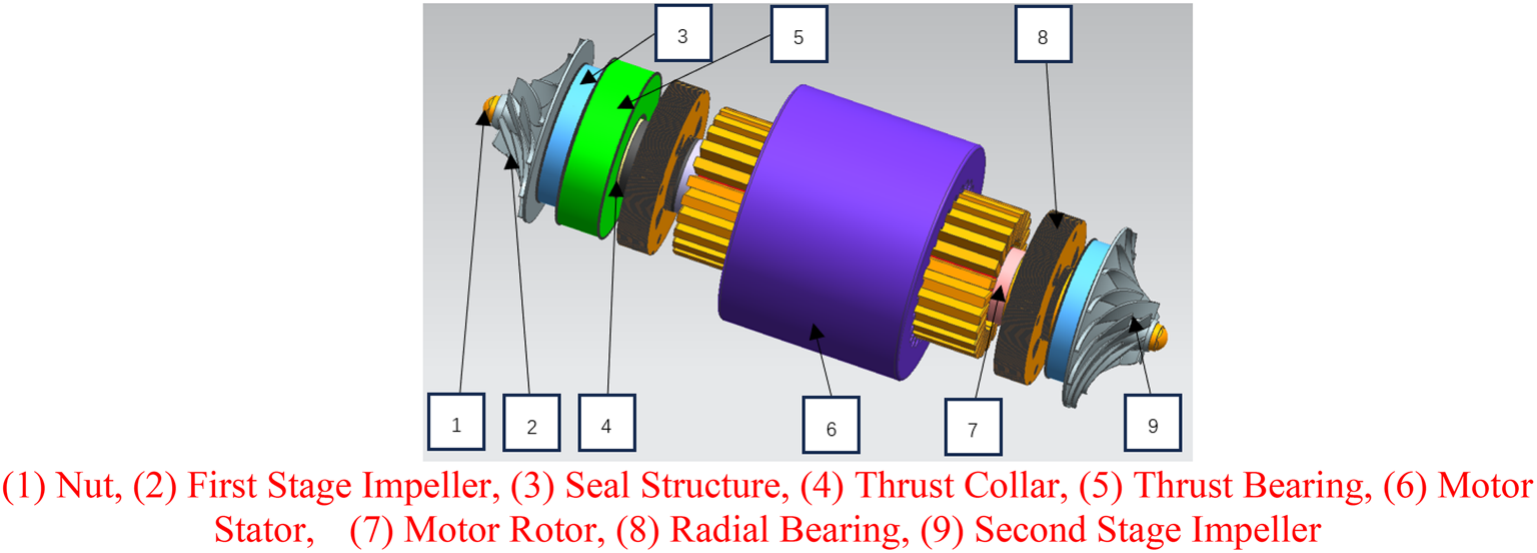
Preliminary three-dimensional model of the compressor.

A preliminary three-dimensional model of the compressor is shown in Fig. 14.

## Rotor dynamics analysis

**Figure 15:**
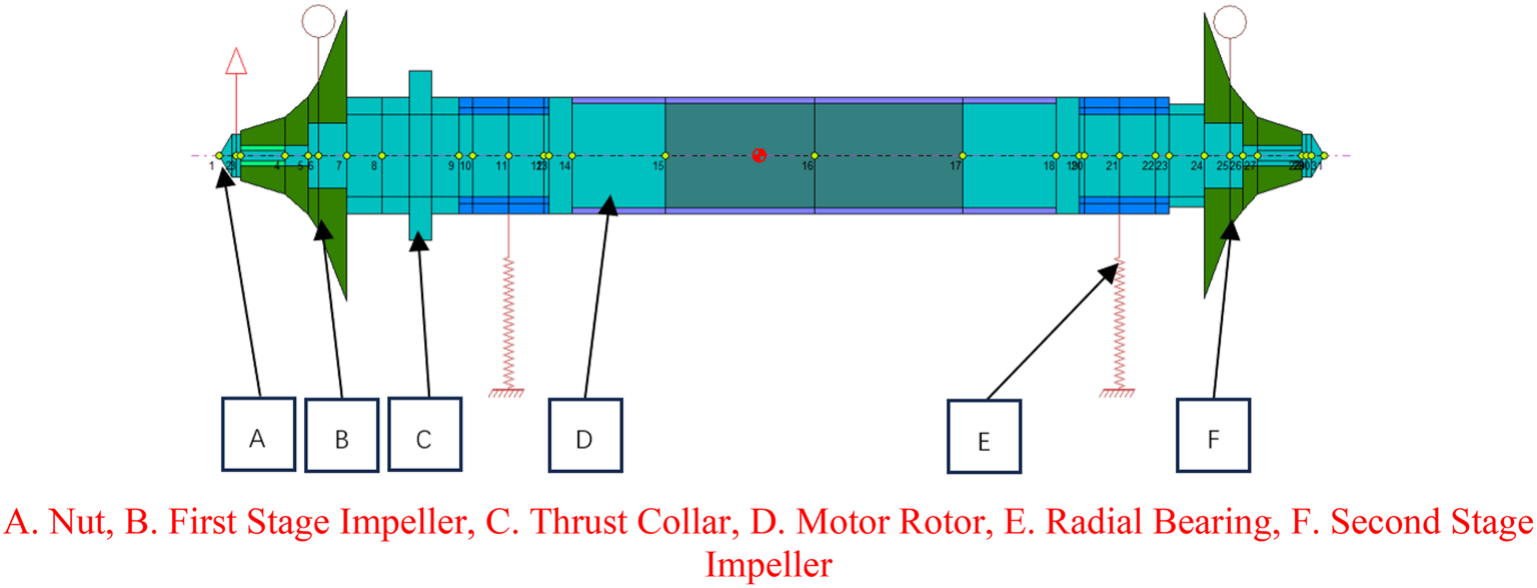
Rotor dynamics model of compressor. A.

The rotor dynamics analysis of compressor rotor is completed by Dyrobes software. The rotor dynamics model is shown in Fig. 15. Shaft elements were used to model the rotor length, and diameter values of nuts, impellers, seal structure, thrust collar and motor rotor. Impellers can be modeled as disks. The mass, diametral moment of inertia and polar moment of inertia of the first-stage impeller are 1.01 kg, 7.198 × 10^−4^m^4^ and 1.328 × 10^−3^m^4^ respectively, and the mass, diametral moment of inertia and polar moment of inertia of the second-stage impeller are 0.88 kg, 6.095 × 10^−4^m^4^ and 1.139 × 10^−3^m^4^ respectively. Both radial bearing and thrust bearing adopt active magnetic bearing, and the initial values of stiffness and damping of radial bearing are set to 1000N/mm and 1N·s/mm respectively.

**Figure 16:**
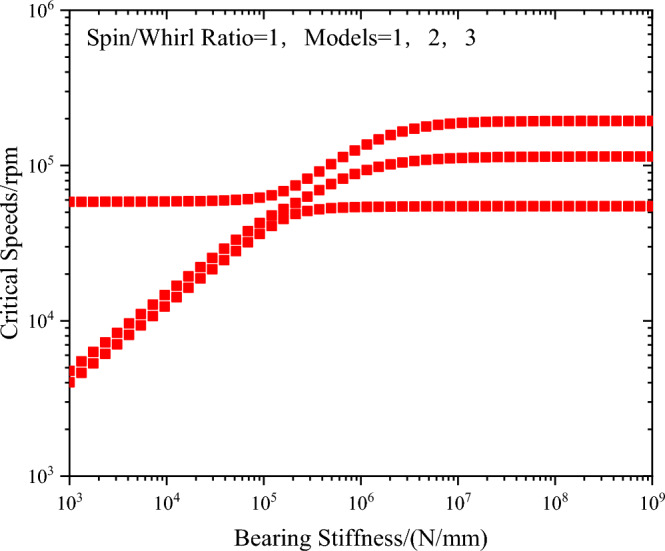
Critical speed map.

The critical speed map of the rotor is shown in Fig. 16. Due to the limitation of the stiffness of magnetic bearing, the critical speed will definitely appear in the operating speed range of carbon dioxide compressor in this study. Therefore, it is necessary to analyze the steady synchronous response of the rotor and further judge whether the compressor rotor is a rigid rotor according to the vibration mode of the rotor.

**Figure 17:**
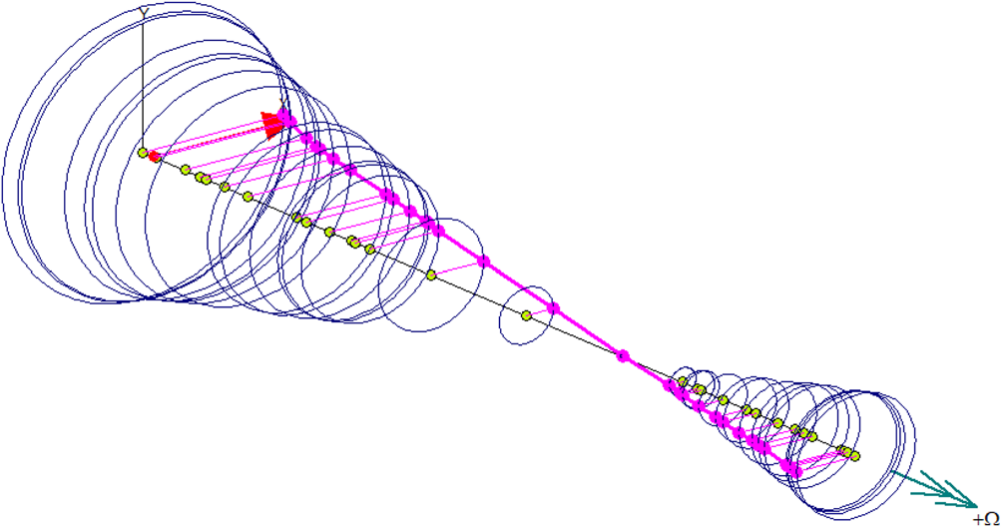
Rotor synchronous response.

The goal of the rotor dynamics analysis was to study the forced unbalance response of rotor and evaluate the stability of the rotor^37^. The rotor synchronous response at the first-order critical speed within the operating speed is shown in Fig. 17 and the results show that the vibration mode of the rotor is oscillating vibration mode and belongs to rigid vibration mode. Therefore, according to API Standard 617, the rotor is a rigid rotor.

**Figure 18:**
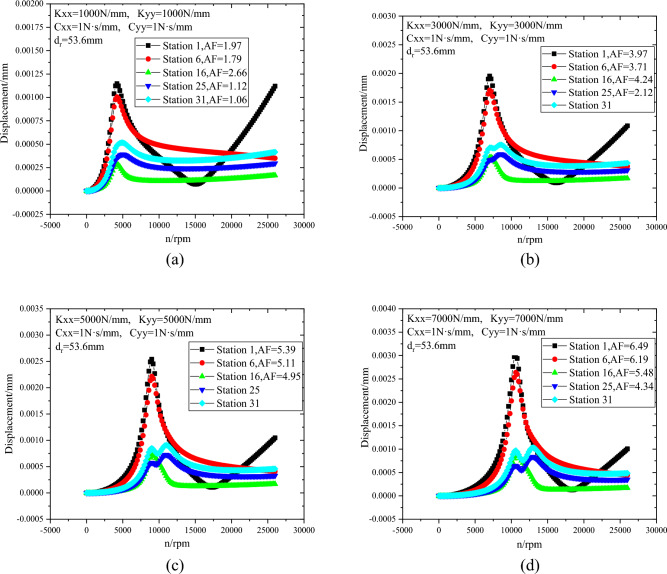
Lateral steady state response with different radial bearing stiffness (**a**) K_xx_ = K_yy_ = 1000 mm, (**b**) K_xx_ = K_yy_ = 3000 mm, (**c**) K_xx_ = K_yy_ = 5000 mm, (**d**) K_xx_ = K_yy_ = 7000 mm.

When the radial bearing stiffness ranges from 1000N/mm to 7000N/mm, the lateral steady state response curves of rotor stations 1, 6, 16, 25 and 31 are shown in Fig. 18. With the increase of bearing stiffness, the first-order critical speed and maximum displacement of the rotor increase, and the amplification factor (AF) of each station also increases. And the results show that the first-order critical speed of the rotor is lower than the design speed. This is mainly because the stiffness adjustment range of magnetic bearing is limited (generally lower than 1 × 10^4^N/mm). As shown in Fig. 16, the first-order critical speed of the rotor cannot be greater than the design speed by adjusting the stiffness of magnetic bearing.

**Figure 19:**
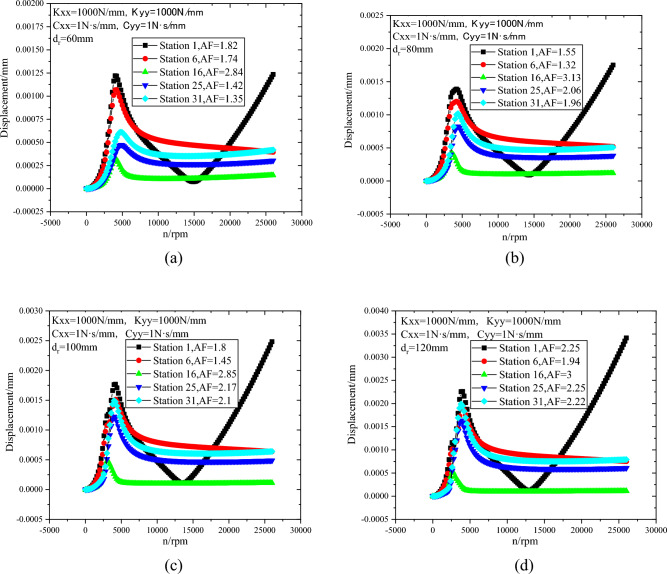
Lateral steady state response with different diameters of motor rotor (**a**) d_r_ = 60 mm, (**b**) d_r_ = 80 mm, (**c**) d_r_ = 100 mm, (**d**) d_r_ = 120 mm.

The lateral steady-state response curves of rotor stations 1, 6, 16, 25 and 31 with different diameters of motor rotor are shown in Fig. 19. When that diameter of the motor rotor is in the range of 60 mm to 120 mm, the maximum displacement of the rotor occurs at the position of Station 1. According to the rotor synchronous response in Fig. 17, the position of Station 1 is the maximum displacement under this vibration mode. With the increase of motor rotor diameter, the maximum displacement of the rotor increases. According to API Standard 617, with the increase of rotor diameter and rotor weight, the allowable unbalance value of the rotor increases, resulting in the increase of the maximum displacement of the rotor. When the motor rotor diameter is 120 mm, the maximum displacement of the compressor rotor is 176.6% higher than that when the motor rotor diameter is 60 mm.

## Results and discussion

In this paper, a centrifugal compressor in carbon dioxide heat pump system is designed. The compressor is directly driven by a high-speed permanent magnet synchronous motor. Two-stage impellers are installed on both sides of the motor, and the bearings are active magnetic bearings. The influences of inlet pressure and temperature on compressor performance are analyzed, the calculation method of compressor axial force is introduced, the axial force is calculated, analyzed and optimized, the rotor dynamics of compressor rotor is analyzed, and the influence of bearing stiffness on rotor dynamics is studied. The conclusions are summarized as follows.

(1) The density of carbon dioxide is greatly influenced by temperature and pressure. When the temperature is 45 °C and the pressure increases from 4 to 6 MPa, the density of carbon dioxide increases by 74.4%. The density of carbon dioxide increased by 20.5% when the pressure was 4.95 MPa and the temperature was increased from 30 to 60 °C.

In the range of inlet temperature from 35 to 55 °C, with the decrease of inlet temperature, the compressor pressure ratio increases by 12–29.8%, the power increases by 2.7–8.6%, and the efficiency decreases more slowly after the design point. In the range of inlet pressure from 4 to 6 MPa, with the increase of inlet pressure, the compressor pressure ratio increases by 12.3–38.6%, and the power increases by 8.7–17.8%. The maximum inlet mass flow of compressor at inlet pressure of 4 MPa is 21.4% lower than that at inlet pressure of 6 MPa.

(2) With the increase of rotating speed, the axial force direction of the first-stage impeller changes from the reverse direction (inlet flow direction) to the positive direction. the axial force varies from  − 1.16kN to 8.51kN when sealing diameter d_m1_ is 30 mm, and from  − 12.14kN to  − 4.79kN when sealing diameter d_m1_ is 60 mm. When the first-stage impeller sealing diameter d_m1_ is constant, the positive value of the axial force of the compressor increases and the negative value decreases when the second-stage impeller sealing diameter d_m2_ ranges from 30 to 60 mm. Through the optimization of the first-stage seal diameter d_m1_ and the second-stage seal diameter d_m2_, it is found that when d_m1_ = 53.6 mm and d_m2_ = 47.2 mm, the absolute value and variation range of the axial force of the compressor are the best.

(3) The vibration mode of the rotor is oscillating vibration mode and the rotor is a rigid rotor. With the increase of bearing stiffness, the first-order critical speed and maximum displacement of the rotor increase, and the amplification factor (AF) of each station also increases. When the motor rotor diameter is 120 mm, the maximum displacement of the compressor rotor is 176.6% higher than that when the motor rotor diameter is 60 mm.

The analysis results in this paper can provide reference for the design of centrifugal compressor in carbon dioxide heat pump system. In the future research work, it is necessary to test and verify the design and analysis results and increase the design power of carbon dioxide heat pump system and centrifugal compressor to ensure their wider popularization and use.

## Data Availability

The datasets generated and/or analysed during the current study are not publicly available due to requirement of confidentiality, but are available from the corresponding author on reasonable request.

## List of symbols

P_0_: Inlet total pressure of compressor (MPa)
p_0_: Pressure in front of the impeller inlet hub (MPa)
p_1_: Inlet pressure of impeller (MPa)
p_2_: Outlet pressure of impeller (MPa)
n: Impeller rotating speed (r/min)
F_1_: Axial force acting on the inlet face of impeller (kN)
${{\text{F}}}_{2}$: Axial force acting on the inlet–outlet part of impeller (kN)
F_1m_: Axial force generated by the change of momentum from axial direction to radial direction (kN)
r_1_: Radius of impeller inlet (m)
d_m_: Diameter of the sealing diameter between the back of the impeller and the motor (m)
D_1_: Diameter of impeller inlet (m)
K_xx_/K_yy_: Stiffness of the journal bearing (N/mm)
F_tot1_: Axial force of first-stage impeller (kN)
d_m__1_: Diameter of the sealing diameter between the back of the first-stage impeller and the motor (m)
d_r_: Diameter of motor rotor (mm)
T_0_: Inlet total temperature of compressor (°C)
F_tot_: Axial force of compressor (kN)
C_01_: Inlet velocity (m/s)
${{\text{Q}}}_{{\text{m}}}$: Mass Flow Rate (kg/s)
D_0_: Diameter of impeller inlet hub (m)
F_0_: Axial force acting on the inlet hub of impeller (kN)
F_3_: Axial force acting on the impeller back (kN)
F_10_: Axial force generated by the static pressure of inlet gas (kN)
r_2_: Radius of impeller outlet (m)
${{\text{F}}}_{\mathrm{tot1,2}}$: Axial force of the first-stage impeller or the second-stage impeller (kN)
D_2_: Diameter of impeller outlet (m)
C_xx_/C_yy_: Damping of the journal bearing(N·s/mm)
F_tot2_: Axial force of second-stage impeller (kN)
d_m__2_: Diameter of the sealing diameter between the back of the second-stage impeller and the motor (m)
